# Altered lumbo-pelvic-hip complex muscle morphometry and contraction change in postpartum pelvic girdle pain and asymptomatic subjects: a cross-sectional study

**DOI:** 10.3389/fphys.2024.1506553

**Published:** 2025-01-06

**Authors:** Ziling Lin, Yanjun Hou, Xueling Chen, Yanping Liu, Xiangbin Wang

**Affiliations:** ^1^ College of Rehabilitation Medicine, Fujian University of Traditional Chinese Medicine, Fuzhou, China; ^2^ Key Laboratory of Orthopedics and Traumatology of Traditional Chinese Medicine and Rehabilitation Ministry of Education, Fujian University of Traditional Chinese Medicine, Fuzhou, China; ^3^ Department of Pelvic Floor Rehabilitation, Affiliated Rehabilitation Hospital of Fujian University of Traditional Chinese Medicine, Fuzhou, China; ^4^ Department of Rehabilitation, Third Affiliated People’s Hospital of Fujian University of Traditional Chinese Medicine, Fuzhou, China

**Keywords:** pelvic girdle pain, postpartum women, lumbo-pelvic-hip complex muscle, musculoskeletal ultrasound, muscle morphometry

## Abstract

**Objective:**

Lumbo-pelvic-hip complex muscle training is considered a crucial component of exercise rehabilitation for postpartum women with pelvic girdle pain (PGP). However, there is a paucity of research evidence regarding the morphological changes and contraction function of these muscles in postpartum women with PGP. Understanding the alterations in lumbo-pelvic-hip complex muscles function associated with PGP, is crucial for tailoring effective rehabilitation strategies and promoting optimal postpartum recovery. Therefore, this study aims to compare the differences in muscle thickness and contraction function of lumbo-pelvic-hip complex muscle between postpartum women with PGP and asymptomatic controls using ultrasound imaging.

**Methods:**

One hundred and fifty postpartum women with PGP and fifty age-matched asymptomatic postpartum women were recruited in this study. Real-time musculoskeletal ultrasound was utilized to measure the resting muscle thickness of nine lumbo-pelvic-hip complex muscles, including the erector spinalis (ES), latissimus dorsi (LD), quadratus lumbalis (QL), gluteus maximus (GMax), gluteus medius (GMed), piriformis (PF), iliacus muscle (IM), rectus femoris (RF), and biceps femoris (BF). The thickness of the GMax, GMed, RF, and BF muscles were measured at maximum contraction to calculate a contraction: rest ratio.

**Results:**

Compared to the asymptomatic women, those with PGP exhibited reduced resting thickness of the ES and GMax muscles bilaterally, as well as increased resting thickness of the right LD and IM muscles. Furthermore, the PGP group demonstrated decreased thickness of the left GMed and right RF muscles at maximum contraction. The contraction ratio was also diminished for the GMax muscles bilaterally, left GMed, and right RF in women with postpartum PGP. Conclusion: Postpartum women with PGP demonstrated reduced thickness, asymmetry, and diminished contractility of the lumbo-pelvic-hip complex muscles. Exercise prescriptions for postpartum women with PGP should incorporate targeted strengthening of the ES, GMax, GMed, and RF muscles.

**Conclusion:**

Postpartum women with PGP demonstrated reduced thickness, asymmetry, and diminished contractility of the lumbo-pelvic-hip complex muscle. Exercise prescriptions for postpartum women with PGP should incorporate targeted strengthening of the ES, GMax, GMed, and RF muscles.

## 1 Introduction

Pelvic Girdle Pain (PGP) is defined as pain located between the posterior iliac crest and gluteal folds, particularly around the sacroiliac joints and/or the pubic symphysis (Vleeming et al., 2008). Studies have shown that the global incidence of pregnancy-related PGP varies from 20% to 65% (Fogarty et al., 2020), with 19% of postpartum individuals continuing to suffer from this chronic condition for up to 11 years postnatally (Elden et al., 2016). For many postpartum women, PGP leads to disability and sick leave, significantly impacting their daily lives (Algard et al., 2023).

The etiology of PGP involves hormonal and biomechanical factors that influence the stability of the lumbopelvic girdle region (MacDonald et al., 2024). Hormonal changes in pregnant and postpartum women significantly impact bone metabolism (Zhao et al., 2024), leading to the softening of connective tissues and increased joint mobility (Casagrande et al., 2015). Furthermore, the progressively enlarging uterus and weight gain during pregnancy contribute to increased loading on the pelvic girdle, resulting in the stretching of the lumbo-pelvic-hip complex muscles and altered pelvic girdle alignment (Katonis et al., 2011).

Previous studies have demonstrated that pelvic girdle stability is maintained through the dynamic interplay between form closure (i.e., fit of bony structures) and force closure (i.e., active muscle support) of the pelvis (Vleeming et al., 2008; Vleeming and Schuenke, 2019). In addition to the deep core muscles (transverse abdominis, diaphragmatic muscle, multifidus, and pelvic floor muscle), the lumbo-pelvic-hip complex muscles, including the erector spinae muscles, gluteus muscles, iliacus muscle, and biceps femoris, have been shown to increase the stiffness of the sacroiliac joint, thereby enhancing pelvic stability (van Wingerden et al., 2004). A reduction in force closure can result in a 32%–68% increase in pelvic joint motion in women with PGP, placing greater demand on the pelvic musculature to accept and transfer load between the trunk and lower extremities (Mens et al., 2009). Therefore, restoring the strength and contractile function of force-closure muscles is an effective approach to improving PGP by increasing pelvic girdle stability.

However, limited evidence exists regarding the effectiveness of general stability exercises for treating PGP during pregnancy and postpartum periods, as demonstrated by two systematic reviews (Almousa et al., 2018; Mapinduzi et al., 2022). The lack of effectiveness may stem from an incomplete understanding of changes in muscle function, and no consensus has been reached on the specific muscle morphology alterations exhibited by PGP patients. Furthermore, deep core muscle training is the most emphasized exercise program in previous studies (Mamipour et al., 2023; Puri et al., 2023), resulting in a paucity of knowledge about the potential relationship between lumbo-pelvic-hip complex muscles morphological changes and PGP. Although these muscles do not directly attach to the pelvic girdle, they are all connected to the thoracolumbar fascia or the posterior ligamentous structure of the sacroiliac joint, contributing to pelvic girdle stability (Bussey and Milosavljevic, 2015). Therefore, understanding the changes in lumbo-pelvic-hip complex muscle morphology and contraction function associated with PGP is essential for developing targeted, comprehensive muscle training programs for postpartum individuals with PGP.

Therefore, the main purpose of this study was to compare the differences in lumbo-pelvic-hip complex muscle between postpartum women with and without PGP. It was hypothesized that postpartum women with PGP would exhibit morphologically thicker lumbar muscles at rest, including the erector spinalis (ES), latissimus dorsi (LD), quadratus lumbalis (QL), piriformis (PF), iliacus muscle (IM), and rectus femoris (RF) muscle. Additionally, reduced muscle thickness and lesser contraction change in the gluteus maximus (GMax), gluteus medius (GMed), and biceps femoris (BF) muscle as measured by ultrasonography, compared with the women without PGP.

## 2 Materials and methods

### 2.1 Participants

**FIGURE 1:**
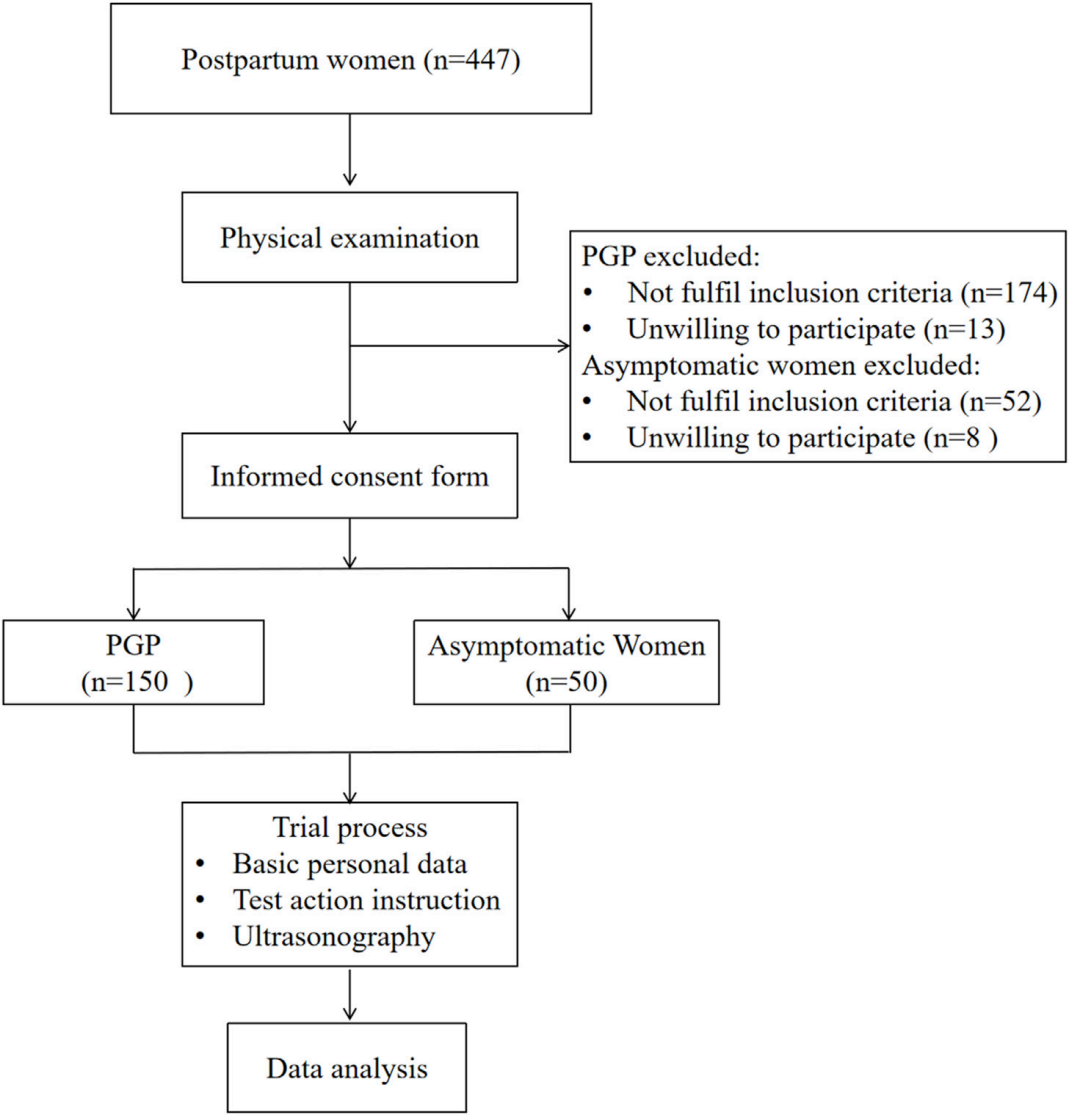
Flow chart of participants through the study.

This cross-sectional study involved a total of 200 postpartum individuals, including 150 participants with PGP and 50 matched controls (Figure 1). Participants were recruited through various methods between March 2023 and May 2024, such as posters and brochures displayed at rehabilitation department in Rehabilitation Hospital and Third People’s Hospital affiliated to Fujian University of Chinese Medicine, newspaper advertisements and referrals from communities near the hospital.

The following inclusion criteria were set for the postpartum women with PGP: (1) fulfilled the updated 2011 diagnostic criteria for PGP, which specify pain in either the posterior pelvic girdle at the sacroiliac joint or pubic symphysis on the anterior side of the pelvic girdle (Kanakaris et al., 2011); (2) postpartum women, 20–40 years of age; (3) within 6 months to 5 years after delivery; (4) a Visual Analogue Scale (VAS) pain score of 3–6 cm; and (5) voluntary participation in this trial and provision of informed consent. The exclusion criteria for the PGP group were: (1) pain located between the subcostal and fifth lumbar regions (Casagrande et al., 2015); (2) symptoms associated with pelvic girdle pain already present before delivery; (3) previous surgery on the lumbar spine, pelvic girdle, hip, or other relevant areas; and (4) acute pelvic inflammation, obvious physiological defects, major diseases, or cognitive impairment. In contrast, the asymptomatic group had: (1) no history of prior PGP; (2) no current pain in the lumbopelvic region for at least 6 months; (3) no multiple joint pain in the extremities; and (4) normal gait pattern. All histories, physical examinations, and outcome assessments were performed by the same physiotherapist. Informed written consent was obtained from participants after explaining the detailed procedure. This study was approved by the Research Committee of the Rehabilitation Hospital affiliated with Fujian University of Chinese Medicine (Fujian, China) (ethics approval number: 2023KY-001-01).

### 2.2 Data collection

**FIGURE 2:**
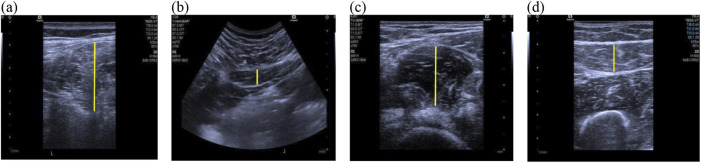
Ultrasonographic Images of muscles at rest. **(A)** Erector spinalis; **(B)** Quadratus lumbalis; **(C)** Iliac muscle; **(D)** Rectus femoris; the yellow lines represent the measurement range of muscles thickness.

Musculoskeletal ultrasound imaging was performed using a Siemens-Sequoia Silver ultrasound system. An experienced physical therapist specializing in women’s health, with over 3 years of experience in musculoskeletal ultrasound conducted the assessments without knowing the participant group. A water-soluble transmission gel was applied to the measurement site, and either the 10L4 (superficial probe) or 5C1 (convex array probe) was placed on the muscle based on the specification outlined in Table 1 (Othman et al., 2023; Nojiri et al., 2021; Taniguchi et al., 2015). Figure 2 illustrates two-dimensional ultrasound imaging of the ES, QL, IM, and RF muscle at rest.

**TABLE 1:** Measurement positions of lumbo-pelvic-hip complex muscles.

Muscle	Positions	Measurement sites
Erector spinalis	Prone	Lateral multifidus muscle of the L5 vertebra, between the transverse process of the lumbar spine and fascia
Latissimus dorsi	Prone	T12 lateral spinous process, the shallowest muscle layer outside the lumbo-iliocostalis muscle
Quadratus lumbalis	Prone	The long axis of probe is perpendicular to the direction of iliac crest, the lateral transverse process of the fifth lumbar vertebra
Gluteus maximus	Prone	30% proximal between posterior superior iliac spine and the greater trochanter
Gluteus medius	Lateral	Midway between the proximal end of iliac crest and the greater trochanter
Piriformis	Prone	the intersection of a line extending from the greater trochanter to the ipsilateral posterior superior iliac spine and a second line extending from the ischial tuberosity and the ipsilateral anterior superior iliac spine
Iliacus muscle	Supine	a level 4 cm medial side of the anterior superior iliac spines
Rectus femoris	Prone	Midway between anterior superior iliac spine and proximal end of patella
Biceps femoris	Prone	Midway between ischial tuberosity and lateral condyle of tibia

Lumbar muscle (ES, LD, QL) thickness were measured at rest on both sides, comparing the symmetry between left-right side. Given that the skin folds and movement characteristics of these muscles during maximum contraction in the prone position influence the stability of the probe, measurements were only obtained at rest. Pelvic gluteal muscles thickness and left-right symmetry (GMax and GMed) were collected at rest and maximum contraction (MC), while PF muscle thickness was only measured at rest due to the difficulty in obtaining accurate measurements at greater depths during maximum contraction. Hip muscles (IM, RF, and BF) thickness were also measured at rest and maximum contraction on the right side (dominant leg). Given that hip flexion could affect probe stabilization, IM muscle thickness was only captured at rest. Each measurement was repeated three times, and calculate the mean value of resting or maximum contracted muscle thickness. Left-right symmetry (L/R) was determined by comparing muscle thickness at rest between the left and right sides. An L/R ratio closer to 1 indicates greater symmetry, while a ratio >1 suggests greater muscle thickness on the left side, and a ratio <1 indicates greater muscle thickness on the right side. The muscle contraction ratio during maximum contraction was calculated using the following equation (Bashir et al., 2019). A higher contraction ratio (CR) indicates better contraction activity.

$$\text{contraction ratio }\left(\text{CR}\right)=\left(\frac{\;{\text{thickness}}_{\text{muscle}\;\text{contracted}}—{\text{thickness}}_{\;\text{rest}}}{{\text{thickness}}_{\;\text{rest}}}\right)$$

### 2.3 Statistical analysis

The analysis was conducted using SPSS (Version 27). Data normality was assessed using the Shapiro-Wilk test and Q - Q plots. Continuous variables were reported as mean (SD) or median (first quartile [Q1]−third quartile [Q3]). Student’s t-test or Mann–Whitney *U* test for continuous data and the chi-square test for categorical data were employed to compare baseline characteristics between the two groups. To compare outcomes in lumbo-pelvic-hip complex muscles thickness and contraction ratio between the two groups, Student’s t-test or Mann–Whitney *U* test was used. Chi-square test was used to compare the qualitative data, i.e., number and mode of delivery. *P* < 0.05 was considered statistically significant across the entire statistical analysis.

## 3 Results

### 3.1 Participants

A total of 200 participants completed the trial, with a mean age of 32.29 years (SD = 4.65). Table 2 lists the clinical characteristics. Both groups were comparable in terms of baseline characteristics. The majority of postnatal women in both groups had delivered once (PGP: 57%, controls: 46%), and most had undergone vaginal deliveries (PGP: 67%, controls: 62%). In the PGP group, pain was predominantly located on the left side (49%), followed by pain in multiple sides (25%), the anterior pelvic region (14%) and the right side (R: 12%). The mean duration of pain in the PGP group was 12.68 months (SD = 9.82).

**TABLE 2:** Between-group differences in baseline characteristics.

Characteristics	PGP (n = 150)				Asymptomatic women (n = 50)				Effect size	*P*
Age, mean (SD), y	32.81 (3.38)				33.40 (2.89)				0.58 (1.64–0.47)	0.27
Height, mean (SD), cm	1.60 (0.55)				1.60 (0.58)				0.00 (0.01–0.02)	0.86
Weight, Me (IQR), kg	56.55 (52.88–62.13)				57.70 (52.73–61.00)				0.00 (0.200–2.30)	0.91
BMI, Me (IQR), kg/m^2^	22.04 (20.32–24.22)				22.16 (20.70–23.48)				0.02 (0.84–0.83)	0.97
Number of deliveries, n	1	2	3	4	1	2	3	4	2.70	0.44
	86	53	10	1	23	23	3	1		
Postpartum time, Me (IQR), m	15.45 (7.76–32.74)				18.00 (6.88–39.18)				2.07 (−7.53 to 2.23)	0.36
Maximum gestational weight, Me (IQR), kg	66.00 (61.00–74.00)				61.75 (61.75–72.35)				0.00 (2.20–3.50)	0.76
gestational age, Me (IQR), day	273.50 (267.75–280.00)				275.00 (272.50–280.00)				1.00 (−4.00 to 1.00)	0.32
Delivery mode, n	1	2		3	1	2		3	0.58	0.75
	101	45		4	31	17		2		
Pain side, n	L	R	AP	Mult	_				_	_
	74	18	21	37						
Disease duration, mean (SD), m	12.68 (9.82)				_				_	_
VAS, mean (SD), cm	4.5 (0.59)				_				_	_

BMI, Body mass index.

Delivery mode, 1; vaginal delivery; 2, cesarean delivery; 3, both 1 and 2.

left side, R; right; AP, anterior pelvic; Mult, At least two areas.

year; m, month; n, number.

IQR, interquartile range; Me, median.

### 3.2 Lumbar and hip muscles morphometry

Table 3 presents the between-group differences in ES, LD, QL, PF, IM, RF, and BF muscle morphometry between postpartum women with PGP and asymptomatic women. The PGP group exhibited lower ES muscle thickness bilaterally (*P*< 0.001). The LD muscle was thicker on the right side and asymmetrical in the PGP group (*P<* 0.05). There were no differences in QL and PF muscle thickness bilaterally or in bilateral symmetry between the two groups (*P*> 0.05). Regarding lower limb muscle morphometry, a statistically significant difference was noted in the right-side IM muscle thickness (*P* = 0.02). Furthermore, the PGP group demonstrated reduced RF muscle thickness at maximum contraction (*P*< 0.001) and a smaller muscle contraction ratio (*P* = 0.01). However, there were no differences in the BF muscle thickness at rest, maximum contraction, and muscle contraction ratio (*P*> 0.05).

**TABLE 3:** Between-group differences in Erector spinalis muscle, Latissimus dorsi Muscle, Quadratus lumbalis, Piriformis, Iliopsoas, Rectus femoris, and Biceps femoris muscle morphometry.

Muscle (mm)	Parameters	PPGP (n = 150)	Asymptomatic women (n = 50)	β (95% CI)	*P*
ES	L	28.36 (2.37)	29.87 (3.39)	−1.52 (−2.37 to −0.66)	<0.001*
	R	29.81 (3.39)	31.63 (3.38)	−1.82 (−2.71 to −0.93)	<0.001*
	L: R	0.95 (0.92–0.97)	0.96 (0.89–0.98)	0.00 (−0.02 to 0.02)	0.83
LD	L	1.21 (0.82–1.56)	0.90 (0.65–1.54)	0.16 (−0.02–0.33)	0.07
	R	1.73 (1.23–2.32)	1.16 (0.78–1.75)	0.49 (0.26–0.73)	<0.001*
	L: R	0.74 (0.54–0.98)	0.85 (0.70–0.99)	−0.10 (−0.20 to −0.02)	0.02*
QL	L	11.55 (2.91)	12.12 (3.42)	0.41 (1.45–0.57)	0.38
	R	11.61 (3.08)	12.15 (3.65)	0.56 (1.68–0.52)	0.31
	L: R	1.03 (0.27)	1.04 (0.29)	0.03 (0.12–0.06)	0.57
PF	L	18.40 (14.80–20.33)	18.62 (15.88–22.34)	−1.17 (−2.70 to 0.31)	0.12
	R	18.35 (4.00)	18.39 (15.67–23.04)	−0.85 (−2.38 to 0.52)	0.25
	L: R	1.00 (0.89–1.07)	1.01 (0.95–1.07)	−0.01 (−0.05–0.24)	0.43
IM	R	15.40 (2.40)	14.51 (2.16)	0.39 (0.12–1.65)	0.02*
RF	R	9.91 (1.90)	10.35 (1.62)	−0.44 (−1.03 to 0.15)	0.14
	MC	13.92 (2.44)	14.97 (14.32–16.28)	−1.33 (−2.08 to −0.67)	<0.001*
	CR	0.39 (0.26–0.57)	0.52 (0.25)	−0.10 (−0.17 to −0.03)	0.01*
BF	R	24.13 (21.13–28.50)	26.20 (23.07–28.93)	−1.77 (0.90–0.01)	0.05
	MC	29.10 (5.43)	30.87 (5.81)	−1.61 (−3.34 to 0.11)	0.07
	CR	0.16 (0.06–0.33)	0.20 (0.32)	−0.01 (−0.11 to 0.08)	0.77

ES, erector spinalis; LD, latissimus dorsi; QL, Quadratus lumbalis; PF, piriformis; IM, iliacus muscle; RF, rectus femoris; BF, Biceps femoris.

* = statistically significant (*p* < 0.05).

### 3.3 Pelvic gluteal muscles morphometry

**FIGURE 3:**
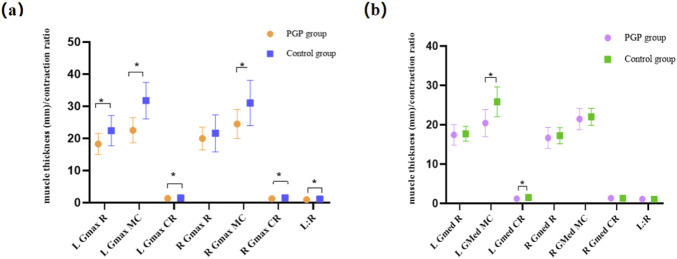
**(A)** Between-group differences in the Gluteus maximus (GMax) muscle thickness and contraction ratio. Figure 3 **(B)** Between-group differences in the gluteus medius (GMed) muscle thickness and contraction ratio. The vertical axis is muscle thickness (mm) and contraction ratio, using the mean and standard deviation of each parameter of the two groups. The horizontal axis is muscle parameters, including the muscle thickness at the rest state (R) and maximum contraction state (MC), contraction ratio (CR), and left-right symmetry at the rest state (L:R). *: Differences existed between groups.

Figure 3A illustrates the muscle thickness and contraction ratio differences in the GMax. The PGP group exhibited significantly reduced muscle thickness at rest, maximum contraction, and muscle contraction ratio on the left side (*P<* 0.05). Moreover, the right GMax muscle thickness at maximum contraction and muscle contraction ratio were significantly lower in the PGP group compared to asymptomatic women (*P<* 0.05). Statistically significant difference was also observed in the left–right symmetry (*P*< 0.001). However, there was no difference in the right GMax muscle at rest (*P* = 0.17).

Figure 3B presents the differences in GMed muscle morphometry between the two groups. There were no differences in the resting muscle thickness on the left and right side (*P*> 0.05). However, the left muscle thickness at the maximum contraction and the muscle contraction ratio were significantly lower in women with PGP compared to asymptomatic women (*P<* 0.05). The right side of GMed muscle thickness at rest, maximum contraction, and contraction ratio did not exhibit differences between the two groups (*P*> 0.05).

## 4 Discussion

To our knowledge, this study is the first to comprehensively examine the changes in lumbo-pelvic-hip complex muscle morphometry in postpartum women with PGP using ultrasound imaging. Compared with asymptomatic women, those with PGP exhibited reduced ES and GMax muscle thickness on both sides. The IM muscle showed more thicker than the control group. Additionally, the PGP group showed an imbalance of LD and GMed muscle activity between the two sides. These findings are partially consistent with the original hypothesis, and provide recommendations for personalized exercise prescription in postpartum women with PGP.

### 4.1 Lumbar muscles

Our findings revealed that women with PGP had reduced ES on both sides compared to asymptomatic women, while the LD muscle was thicker than the asymptomatic women and more thicker on the right side. To our knowledge, no previous study has specifically examined ES and LD muscle morphology in this specific population, resulting in a lack of direct comparative analyses. In functional anatomy, these muscles cross over to produce movement via attachments at the spine or hip (Fiani et al., 2021). Moreover, the ES, BF, and GMax muscles have the greatest effect on sacroiliac joint stiffness (Farley et al., 2024). In this study, the postpartum PGP group exhibited reduced muscle thickness of ES on both sides, potentially indicating a decreased ability in the force closure function of the pelvis. The current results were similar with the findings of Ronchetti et al. (2008) who used muscle strength test to demonstrate that postpartum women with PGP exhibited reduced strength and endurance of the trunk flexors and extensors compared with pain-free postpartum women. Shadmehr et al. (2012) found a smaller recruitment of ES during active straight leg raise (ASLR) in women with sacroiliac pain compared to controls, while their study participants were not pregnant or postpartum women. In the current study, the LD muscle was thicker in the PGP group. A study in healthy volunteers demonstrated that excessive LD activity during ASLR could be induced by experimental pelvic pain (Palsson et al., 2015). The increased LD muscle thickness in the PGP group observed in this study might be a compensatory action for trunk extensor insufficiency, aiming to maintain pelvic stability during childcare activities. However, due to the limited number of studies on lumbar muscles morphometry in postpartum individuals with PGP, conclusions remain tentative, and further research is necessary to elucidate these findings.

Furthermore, no significant differences in PF and QL muscle thickness were observed between the two groups. This may be attributed to changes in muscle tone rather than thickness. Wójcik, M et al. (2023) using a review of journal databases, found that increased lumbar and PF muscle tone was strongly associated with lumbopelvic pain. Therefore, it is very important to relax the PF and lumbar muscle. Additionally, the PGP group exhibited greater asymmetry, with the right LD muscle demonstrating increased thickness compared to the left side. This finding contrasts with the results of Bashir et al. (2019), who reported a significant decrease in LD muscle thickness when compared to the painful side in subjects with sacroiliac joint dysfunctions. While in this present study, a tendency for LD muscle thickening on the contralateral side of the painful area (predominantly on the left side in PGP group, 49%). These discrepancies may be partially attributed to participant selection, as their study did not focus specifically on postpartum women. Anatomically, in the posterior oblique chain, the GMax is connected via fascia to the sacroiliac joint and the contralateral LD (Gibbons, 2017). As most subjects in this study experienced left-sided pain, muscle spasms may inhibit the GMax on the painful side, potentially leading to compensatory hypertrophy of the LD muscle on the non-painful side. In summary, ES muscle strengthening and LD lengthening should be considered when developing individualized exercise prescriptions for postpartum PGP.

### 4.2 Pelvic gluteal muscles

In this study, the PGP group displayed thinning in bilateral GMax and a lower contraction ratio on both sides compared with asymptomatic women. Additionally, left-right asymmetry of muscle at rest was observed in the PGP group. This result is similar with previous reports by Bashir et al. (2019), which found that GMax muscle thickness decreased significantly among subjects with sacroiliac joint dysfunctions on the pain side. Bagwell et al. (2020) demonstrated that the GMax plays a crucial role in pelvic force closure and dynamic stability of the low back and pelvis, and disuse of the GMax muscle could increase joint loads and potentially contribute to pain during and after pregnancy. Furthermore, Shadmehr et al. (2012) utilized electromyography (EMG) and observed a significant difference in tonicities of the BF, GMax, and ES muscles between patients with sacroiliac joint dysfunction and healthy controls. Ludwig et al. (2024) found that the GMax muscle had the strongest correlation with changes in pelvic tilt in healthy individuals. Moreover, the GMax muscle is morphologically densely linked to the pelvic floor via strands of connective tissues investing the adjacent muscles, which also has an impact on pelvic floor dysfunction (Siess et al., 2023). In the current study, postpartum individuals with PGP exhibited notably reduced GMax muscle thickness on both sides compared to asymptomatic women, suggesting potential laxity in muscle contraction function. As reduced muscle thickness might affect the biomechanical properties of the joint by altering mechanical stress and load transfer (Bashir et al., 2019), it may lead to the occurrence of chronic pain. Therefore, it is recommended to emphasize GMax muscle strength in exercise training for this population. Further investigation of its morphology and activation during tasks is also essential.

Regarding the GMed muscle, the PGP group exhibited reduced muscle thickness at maximum contraction and a lower contraction ratio on the left side. The findings of this study is similar with previous research conducted on patients with low back pain and individuals with no history of pain. Aboufazeli et al. (2018) employing the same ultrasound method, observed a reduction in GMed muscle thickness change in patients with low back pain. Similarly Cooper et al. (2016), using manual muscle testing, discovered that GMed muscle weakness was a common symptom in participants with chronic non-specific low back pain. In conjunction with the GMax muscle results, the left gluteus muscle was suppressed, and contractile dysfunction was more pronounced in patients with PGP. Considering the crucial role of GMed muscle as a pelvic stabilizer during gait and its strong correlation with musculoskeletal dysfunction diseases (Fenato et al., 2021), strengthening the GMed muscle on the painful side is a recommended protocol for exercise prescription in postpartum women with PGP. Furthermore, future research investigating the influence of PGP on gait biomechanics, utilizing motion analysis and EMG systems, is warranted.

### 4.3 Hip muscles

Regarding the lower limb muscles, the iliacus muscle thickness on the right side was significantly greater in the PGP group compared to the control group. To our knowledge, this study is the first to examine the characteristic changes of the iliacus muscle in postpartum individuals with PGP. Vleeming et al., 's 2008 pelvic girdle guidelines recommend palpating the iliopsoas muscle in patients with PGP, but do not provide specific characteristics (Vleeming et al., 2008). Anatomically, the iliacus muscle joins the psoas muscle to form the iliopsoas muscle, which is surrounded by the dense iliac fascia (Siccardi et al., 2024). The fascia covering the iliopsoas muscle forms multiple fascial connections, linking the muscle to various viscera and muscle regions (Bordoni et al., 2023). Our findings regarding the iliacus muscle is similar with the work of Fujitani et al. (2021), who discovered higher EMG activity in the iliacus muscle of participants with recurrent lower back pain compared to healthy controls. Clinically, iliac muscle stiffness can often be detected through palpation. Using ultrasonography Miyachi et al. (2021), found a significant positive correlation between anterior pelvic tilt position and iliopsoas muscle thickness. Based on these findings, we hypothesize a potential relationship between changes in iliac muscle thickness and increased pelvic tilt. Radiographic studies have indicated that individuals with sacroiliac joint pain often exhibit increased anterior pelvic rotation during ASLR compared with healthy individuals (Mens et al., 1999). Future study could incorporate pelvic imaging methods to analyze the relationship between pelvic tilt angle and iliac muscle characteristics in postpartum women with PGP.

In term of the RF and BF muscle, our results showed that the RF muscle thickness at maximum contraction and contraction ratio were significantly smaller in the PGP group than in the asymptomatic group, while no significant differences were observed in the BF muscle morphometry between the two groups. Anatomically, the RF muscle originates from the anterior inferior iliac spine and inserts into the patellar ligament above the patella. The RF muscle is connected to the pelvis via the anterior inferior iliac spine, and its contraction and relaxation have an effect on the position and movement of the pelvis. Tightness or shortening of the RF muscle may lead to anterior pelvic tilt, increasing lumbar lordosis and sacral tilt angle, potentially causing low back pain and pelvic girdle pain (Gibbons, 2017). In combination with other results from this study, postpartum women with PGP may exhibit tense contraction of the iliac muscle while the RF muscle may be inhibited due to pain. A previous study by Yoo (2013) demonstrated that strengthening exercises targeting anterior pelvic tilt muscles (ES, IM, RF) can restore pelvic tilt angles. Therefore, exercise training for individuals with PGP should also focus on strengthening the contraction ability of RF muscle.

Although the current study did not find any difference in the BF muscle, previous research using EMG methodology had different findings regarding BF muscle activity. Shadmehr et al. (2012) observed reduced recruitment of the BF muscle during the ASLR test in women with sacroiliac pain compared to healthy controls. Conversely Palsson et al. (2015), noted excessive BF muscle activity in healthy volunteers during an experimental pain-inducing ASLR test. Two studies by Bussey and Milosavljevic (2015); Bussey et al. (2020), they reported that patients with PGP appear to employ a symptom-led strategy for bracing the innominate through opposing tension in the BF muscle and external oblique during movement of the affected side, potentially increasing mechanical stress on the sacroiliac joint and exacerbating pain. And patients with PGP exhibited longer muscle onset latencies in the BF muscle under visual occlusion (Bussey et al., 2020). While these studies did not focus on postpartum women, the significant impact of BF muscle activity on lumbopelvic control cannot be disregarded. Moreover, a prospective study demonstrated that BF muscle delay during single-leg lift in pregnancy was a significant predictor for the development of PGP in late pregnancy (Aldabe et al., 2020). Therefore, further investigation on the influence of PGP on BF muscle activity using EMG systems in postpartum women is warranted.

### 4.4 Limitations of the study

This study has several limitations that should be acknowledged. First, the research included only individuals with mild to moderate PGP. Future studies could explore whether those with severe PGP exhibit different performance characteristics. Second, due to the limited number of studies investigating muscle morphometry and contraction changes in the LD, IM, RF and BF muscle of postpartum women with PGP, the conclusions about these muscles remain tentative. Third, some muscle thickness measurements were only collected at rest. Future research could incorporate EMG method to better observe muscle changes during various functional tasks, and what sort of the muscle asymmetry associated with pain might also be warranted.

## 5 Conclusion

The current study found that participants with PGP exhibited differences in lumbo-pelvic-hip complex muscle morphometry. Our results indicated that postpartum women with PGP exhibited altered lumbo-pelvic-hip complex muscle morphometry and reduced contraction change. These women with PGP presented reduced ES, GMax, GMed, and RF muscle thickness, thicker LD and IM thickness, and smaller contraction change in the GMax muscle on both sides and GMed muscle on the left side. The current findings provide a better understanding of the impact of PGP in postpartum women and offer a rationale for designing effective exercise interventions.

## Data Availability

The raw data supporting the conclusions of this article will be made available by the authors, without undue reservation.
